# Tagmatization in Stomatopoda – reconsidering functional units of modern-day mantis shrimps (Verunipeltata, Hoplocarida) and implications for the interpretation of fossils

**DOI:** 10.1186/1742-9994-9-31

**Published:** 2012-11-14

**Authors:** Carolin Haug, Wafaa S Sallam, Andreas Maas, Dieter Waloszek, Verena Kutschera, Joachim T Haug

**Affiliations:** 1 Zoological Institute and Museum, Department of Cytology and Evolutionary Biology, University of Greifswald, Soldmannstr. 23, 17487, Greifswald, Germany; 2 Department of Marine Science, Suez Canal University, Ismailia, 41522, Egypt; 3 Biosystematic Documentation, University of Ulm, Helmholtzstr. 20, 89081, Ulm, Germany

**Keywords:** Tagmata, Evolution, Unipeltata, Macro-fluorescence, Sclerites

## Abstract

**Introduction:**

We describe the tagmatization pattern of the anterior region of the extant stomatopod *Erugosquilla massavensis*. For documentation we used the autofluorescence capacities of the specimens, resulting in a significant contrast between sclerotized and membranous areas.

**Results:**

The anterior body region of *E. massavensis* can be grouped into three tagmata. Tagma I, the sensorial unit, comprises the segments of the eyes, antennules and antennae. This unit is set-off anteriorly from the posterior head region. Ventrally this unit surrounds a large medial sclerite, interpreted as the anterior part of the hypostome. Dorsally the antennular and antennal segments each bear a well-developed tergite. The dorsal shield is part of tagma II, most of the ventral part of which is occupied in the midline by the large, partly sclerotized posterior part of a complex combining hypostome and labrum. Tagma II includes three more segments behind the labrum, the mandibular, maxillulary and maxillary segments. Tagma III includes the maxillipedal segments, bearing five pairs of sub-chelate appendages. The dorsal sclerite of the first of these tagma-III segments, the segment of the first maxillipeds, is not included in the shield, so this segment is not part of tagma II as generally thought. The second and third segments of tagma III form a unit dorsally and ventrally. The tergites of the segments of tagma III become progressively larger from the anterior to the posterior, possibly resulting from a paedomorphic effect during evolution, which caused this reversed enlargement.

**Conclusions:**

The described pattern of tagmosis differs from current textbook knowledge. Therefore, our re-description of the anterior body area of stomatopods is of considerable impact for understanding the head evolution of Stomatopoda. Likewise, it has a bearing upon any comparisons with fossil stomatopods, as mainly sclerotized areas are fossilized, and, on a wider scale, upon larger-scale comparisons with other malacostracans and eucrustaceans in general.

## Introduction

Malacostraca includes all the more commonly known and larger crustacean species with a more strongly calcified cuticle (e.g., shrimps, crayfish, crabs, wood lice). Among them the mantis shrimps (Stomatopoda) are probably the most impressive representatives. This is due to their possession of astonishing morphological features such as their extremely complex visual system [1,2] or their fearsome raptorial appendages [3]. Extant mantis shrimps are thus highly derived as compared to other eumalacostracans.

Their unusual morphology is, however, partly bridged by a number of fossil representatives [4] and relatives from the Carboniferous about 360 million years ago [5-8], from the Jurassic (about 150 mya) and also the Cretaceous (about 90 mya) [9-14]. Along the early evolutionary lineage towards the modern representatives of mantis shrimps (Verunipeltata [13]), changes in morphological structures could be followed in much detail permitting a detailed reconstruction of character evolution [6,13]. Examples of more dramatic changes are those of the maxillipeds and the tail fan, but the general body organization, the tagmatization (subdivision of a segmented body into functional units or tagmata), also did not remain unaltered during evolution [6,13].

The study of tagmatization is an important field of research within arthropod evolutionary biology. The division-of-labor principle has even been assumed to be one of the major factors for the evolutionary success of this animal lineage. Additionally, some of the major (eu)arthropod groups have rather ‘fixed’ patterns of tagmosis, i.e., there is only limited variation of these patterns within their in-groups. Examples are the insects with six head segments, three main appendage-bearing thoracomeres and a posterior body region of ≤ 11 segments, and Malacostraca with six head segments, 14 appendage-bearing segments uniformly divided into an eight-segmented and a six-segmented part (only phyllocarids have one more) [15-17]. Significant tagmatization changes within groups may therefore mark the starting point for adaptive radiations, i.e., represent key novelties of newly diversifying groups.

Tagmatization in fossil arthropods is more difficult to understand, not least because incomplete preservation often prohibits access to important structures. Yet, studying fossils for these aspects is very important as they may exhibit patterns of tagmosis not developed in extant representatives [15,16,18-20]. This may include character states that are ‘intermediate’, and therefore can add important details for understanding the evolution of a certain character along an evolutionary lineage. An example is the head of Euarthropoda. Traditionally, a six-segmented head was assumed to have been present in the ground pattern of Euarthropoda, because at least six head segments occur in extant crustaceans, insects, myriapods and chelicerates (ocular segment plus at least five appendage-bearing segments). Yet, this assumed condition turned out to be untrue for certain crustaceans and all chelicerates. Palaeontological data point to a ground-pattern condition of five (ocular plus four appendage-bearing) segments in the head in the stem species of Euarthropoda; this condition occurs in many fossil euarthropods, such as derivatives of the stem lineages of Chelicerata [21-24], Trilobita [25], naraoiids [26] and early representatives of Crustacea sensu lato [27-30]. Furthermore, it is important to have a clear knowledge of the morphology of extant representatives of a group to provide a sound framework for correctly interpreting the pattern of tagmosis of a fossil representative.

In this paper, we aim at contributing to the understanding of the evolution of tagmatization within Stomatopoda by re-evaluating the tagmosis of extant mantis shrimps. For this purpose, we investigated the external morphology of adults of *Erugosquilla massavensis* (Kossmann, 1880), including the application of state-of-the-art documentary techniques [31-33]. Supplementary studies were conducted on late larval material of *Pseudosquillopsis cerisii* (Roux, 1828), for which dissection was permitted. Our contribution focuses on morphological structures that are to a certain degree probably known to some in-group experts, but we will discuss them in the broader context of the evolutionary history of Stomatopoda. This will facilitate better comparisons with various fossil species in the future, as will be exemplified in part here, but, furthermore, improve comparative studies on a broader scale of, e.g., Malacostraca and Eucrustacea in general.

## Results

**Figure 1:**
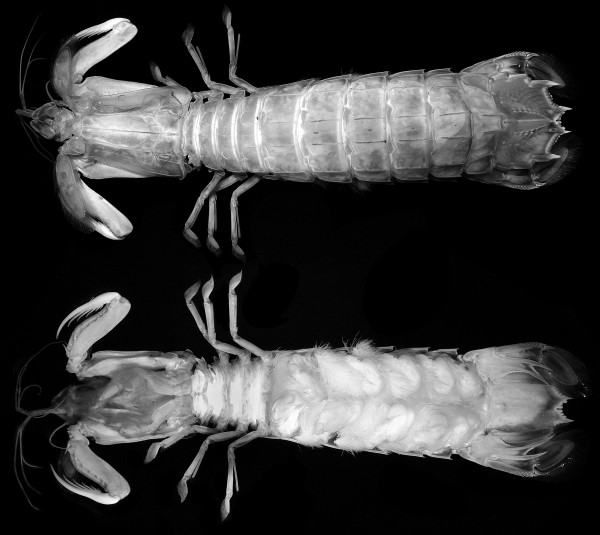
**Overview of *Erugosquilla massavensis *(Kossmann, 1880).** Dorsal (top) and ventral view (bottom). Images taken under crossed polarized light settings [59]. Specimens immersed in alcohol.

The body of the investigated stomatopods (Figure 1) can be grossly subdivided into five functional units or tagmata. These units are identified based on the following criteria:

1) Dorsally or ventrally conjoined segments (e.g., shield)

2) Similar dorsal morphology (mostly tergites)

3) Similar appendage morphology (implying similar function) or related function (but differing morphology, e.g., mouthparts)

4) Close spatial association of segments versus long distance to other segments.

**Figure 2:**
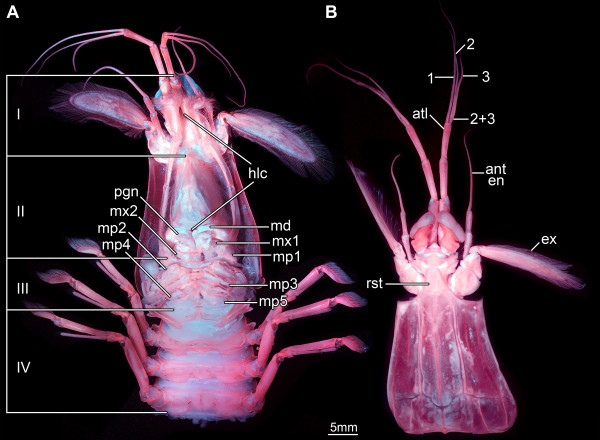
**Principle organization of the anterior body of *Erugosquilla massavensis *(Kossmann, 1880).** Images taken under macro-fluorescence settings. **A**. Ventral view of a specimen, pleon removed. Four major tagmata can be distinguished as described here: I. Sensory area with segments of eyes, antennulae and antennae. II. Feeding area, with elongated posterior part of hypostome (part of hypostome-labrum-complex) and segments of mandibles, maxillulae and maxillae. III. Posterior food-processing unit with segments of maxillipeds 1-4 and the anterior region of the next segment with maxilliped 5. IV. Walking-appendage area; most parts of the segment of maxilliped 5 (except for the appendage itself) being closely associated with the next three segments carrying the walking appendages. **B**. Dorsal view of shield and tagma I (see A) detached from the body. Tagma I is associated with the rostral plate. It comprises the antennulae with three flagella arising in a specific pattern: Flagellum 1 arises from the most distal of the three peduncle elements. An elongated element (2 + 3) inserts adjacent to flagellum 1, which is the common base of flagellum 2 and 3. Abbreviations: I-IV = tagmata I-IV; 1-3 = antennular flagella; ant en = antennal endopod; atl = antennula; ex = exopod; hlc = hypostome-labrum-complex; md = mandible; mp = maxilliped; mx1 = maxillula; mx2 = maxilla; pgn = paragnaths; rst = rostrum.

The five tagmata identified here are the sensorial unit (tagma I), the anterior food-processing unit (tagma II), the posterior food-processing unit (tagma III), the walking-appendage area (tagma IV), and the pleon plus tail fan (tagma V). The anterior three functional units are described here (Figure 2A); tagmata IV and V will be treated in a separate publication (Kutschera et al. in prep.). Descriptions are kept short and general to provide a broader comparability to the fossil forms, but not for differentiating small details that might be important for small-scale taxonomic issues. Remarks are given on structures that differ according to our observation from what has been described in the literature before. Note that the distinction between sclerotized and membranous areas was enabled by the use of autofluorescence, resulting in pink sclerotized and white membranous areas in the figures (for details see Material and methods part below).

### Tagma I: sensorial unit

**Figure 3:**
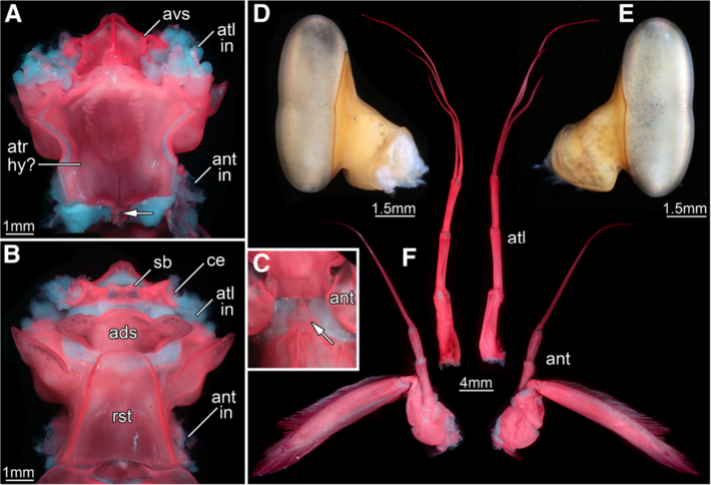
**Details of tagma I of *Erugosquilla massavensis *(Kossmann, 1880). A**-**C** and **F** under macro-fluorescence settings; **D**, **E** under crossed polarized light. **A**. Ventral view of tagma I, appendages and eyes removed. A large central sclerotic plate might represent the anterior part of the hypostome. Antero-laterally the antennulae insert, postero-laterally the antennae insert. Anterior to the possible anterior part of the hypostome another sclerite is present, here referred to as the antero-ventral sclerite, probably associated with the eye segment. The arrow marks the anterior part of a small sclerite posterior to the possible anterior part of the hypostome (see also C). **B**. Dorsal view of tagma I. The rostral plate covers this area and is attached to the posterior region of the segment of the antennae. Between the eyes a small sclerotic bridge is developed. Posterior to this bridge an antero-dorsal sclerite is present, possibly part of the antennular segment. **C.** Close-up of the small sclerite marked by an arrow in A. It is situated right between the antennae. **D**, **E**. Compound eyes. **D**. Median view. **E**. Lateral view. **F**. Antennulae (median) and antennae (lateral); left: anterior view, right: posterior view. Abbreviations other than before: ads = antero-dorsal sclerite; ant = antenna; ant in = antenna insertion; atl in = antennula insertion; atr hy? = supposed anterior part of hypostome; avs = antero-ventral sclerite; ce = compound eye (insertion); sb = sclerotic bridge.

This most anterior tagma is recognized based on a partly conjoined ventral area (criterion 1), a related function of the appendages of its segments (criterion 3), and the close spatial association of its segments (criterion 4). Tagma I includes the three anterior segments, the eye segment (also called ocular somite or ophthalmic somite), the antennular and the antennal segment. It therefore can be termed the sensorial unit. Tagma I is set off from the dorsal shield (traditionally also called carapace, but see [34] for a discussion of the usefulness of this term and alternatives) by a distinct joint, and its posterior region is attached to the rostrum (see below; Figures 2B, 3B). 

The ventral surface of tagma I is made up of three distinct sclerotic cuticular areas. The large most anterior, more or less pentagonal sclerite (here called antero-ventral sclerite, Figure 3A) appears to belong to the eye segment. This antero-ventral sclerite is succeeded posteriorly by a larger, also more or less pentagonal sclerite with one pronounced notch on each side (Figure 3A). It provides the attachment sites of the appendages of the first two appendage-bearing segments, i.e., the antennulae and antennae (Figure 3A), and is therefore interpreted as the anterior part of the hypostome [35-37] (supposed posterior part of the hypostome in tagma II, see below). Between the insertion areas of the antennae, following the supposed anterior part of the hypostome, a small more or less triangular sclerite is situated; it may also be a part of the hypostome (arrows in Figure 3A, C). It touches the posterior rim of the anterior part of the hypostome with one tip and connects the anterior and the posterior part of the hypostome (in tagma II, see there), as it lies directly in the joint area between tagma I and II.

The dorsal area of tagma I comprises three distinct sclerites from anterior to posterior. The anterior sclerite is small, elongate, dumbbell-shaped and bridges the eye stalks (sclerotic bridge in Figure 3B). This bridge is followed by a larger (wider and longer) antero-dorsal sclerite, which is divided into a median sub-trapezoidal part and spatulate tergopleural-like extensions to either side (also called ocular scales; Figure 3B); it is most likely part of the antennular segment (though usually interpreted as part of the eye segment, see below). The antero-dorsal sclerite is followed by an even larger square-shaped sclerite reaching far laterally and ventrally to the antero-lateral rims of the anterior part of the hypostome (Figure 3B, see also Figure 3A); the lanceolate tergopleurae on this sclerite point antero-laterally (Figure 3B). This third dorsal sclerite apparently belongs to the segment of the antennae (in contrast to the traditional interpretation as part of the antennular somite, see below). On top of this sclerite sits the rostral plate (Figure 3B), which is connected so firmly to it that sclerite and rostrum can only be separated by applying quite some force.

The compound eyes are located on the tip of a well-sclerotized stalk. The ommatidia-covered softer surface is organized, in relation to the body orientation, into the typical ventral and dorsal region separated by the slightly constricted midband (Figure 3D, E). The proximal part of the antennulae comprises a peduncle consisting of three tube-shaped consecutive elements. From the most distal element a flagellum arises next to a fourth, much shorter tubular element, on which the two distal flagella insert (Figures 2B, 3F). The antennae have a well-developed, more or less flattened cylindrical coxa and a similarly shaped basipod from which the two rami arise. The endopod extending medio-distally is composed of three tubular elements, the proximal one being rather short, the distal one carrying a long flagellum. The exopod is bipartite: from a small proximal element a much larger paddle-like element arises distally, equipped with many setae along its rim (Figures 2B, 3F).

### Tagma II: anterior food-processing unit

The second tagma is characterized by a dorsally conjoined area (criterion 1), a related function of the appendages of its segments (criterion 3), and a close spatial association of its segments (criterion 4). Tagma II is involved in food handling and intake. Dorsally the tagma forms the sub-rectangular, dorso-ventrally flattened shield (Figure 2B), ventrally an anterior and a posterior region can be distinguished.

**Figure 4:**
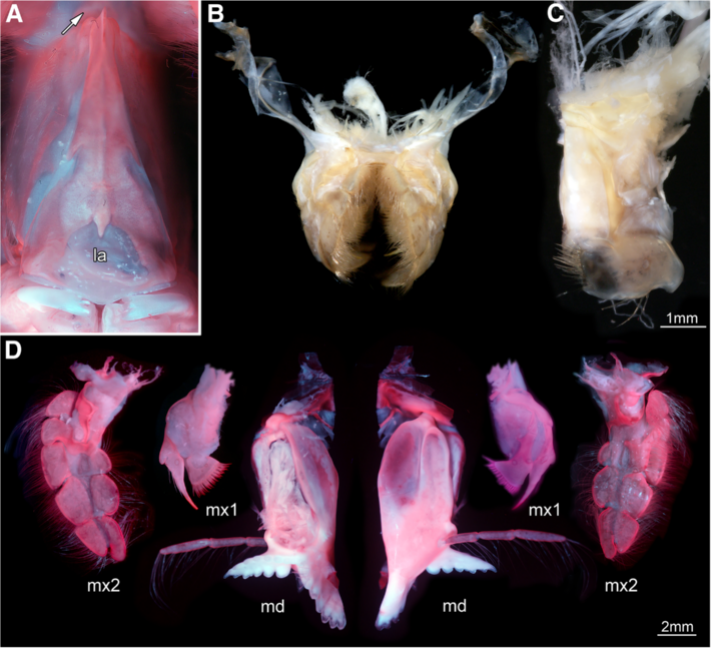
**Details of tagma II of *Erugosquilla massavensis *(Kossmann, 1880). A**, **D** under macro-fluorescence settings; **B**, **C** under crossed polarized light. **A**. Close-up of the supposed posterior part of the hypostome-labrum complex (see also Figure 2A). Arrow marking small sclerite as in Figure 3A and C; not to scale. **B**. Detached post-mandibular feeding apparatus, including maxillulae, maxillae and paragnaths; posterior view; not to scale. **C**. Paragnath; anterior view. **D**. Mandibles, maxillulae, maxillae. Left in anterior view; right in posterior view. Abbreviations as before.

The anterior ventral region, extending to about two thirds of the entire length of the tagma, is completely occupied by a large, elongate triangular, slightly elevated and partly sclerotized structure (Figure 4A). This structure is interpreted as the strongly elongated posterior part of the hypostome (see discussion). The rear end of the hypostome is slightly drawn out into a small lip-like structure overlapping the mouth area, which is interpreted as the labrum (Figure 4A). The mouth opening is located at the rear end of the hypostome. Together with the hypostome, the labrum forms a functional unit, the hypostome-labrum complex.

The posterior region of tagma II is formed by the segments of the mandibles, maxillulae and maxillae. The mandibles lie directly behind the hypostome-labrum complex and are tightly followed by a complex that is composed of the paragnaths, the flat and anteriorly concave maxillulae and the similarly shaped maxillae (Figure 4B). The mandibles consist of a prominent well-sclerotized proximal portion, the coxa, and a distal tripartite palp. The coxa is drawn out medially into the gnathobase with the pars molaris proximally (anteriorly in original topology) and the pars incisiva distally (posteriorly in original topology). Pars molaris and pars incisiva form a 90-degree angle when viewed medially (Figure 4D). The tripartite palp arises medio-distally from the coxa and comprises tubular elements equipped with fine setae (Figure 4D). The paired paragnaths are located between the mandibles (Figure 4C). They are not appendages, but prominent elevations arising from the ventral mandibular sternite [34,35] and form the posterior border of the greater mouth area and an important component of the feeding apparatus [36]. In *E. massavensis* the paragnaths are lobe-shaped and medially equipped with about 20 setae.

The maxillulae are smaller than the mandibles (Figure 4D). They comprise only two elements, the coxa with one median shovel-like endite equipped with about 20 cuspidate setae along its median edge, and the basipod, also with one endite but of spine-like shape and equipped with three smaller cuspidate setae distally. A small hump medio-distally on the basipod could represent the remnant of the endopod.

The maxillae appear to be composed of four elements (Figure 4D), but it is difficult to assign an identity to these elements. A “normal” malacostracan maxilla is composed of a coxa with two median endites and a basipod also with two median endites. It is possible that the four elements seen here correspond to these four endites, but this remains unclear at present. The description is, therefore, kept under open terminology. The most proximal element is not extended into a median endite but has a lateral swelling with a sclerotized outer rim. The second element has a setose enditic lobe medially with a sclerotized edge. A sclerotic bridge on the anterior side of the maxilla connects this enditic part to a small lateral lobe, which is followed more distally by another lateral lobe that is about twice the size of the proximal one. Also this lobe appears to belong to the second element. The lateral lobes are also sclerotized along their outer margins. The distal two elements of the maxillae are both composed of a median and a lateral lobe. All lobes bear setae and are sclerotized along their free margins, leaving the middle of the anterior and posterior area of the maxillae unsclerotized and soft (Figure 4D).

### Tagma III: posterior food-processing unit

Tagma III behind the maxillary segment is characterized by a similar morphology of the appendages of its segments (criterion 3) as well as a similarity of the dorsal morphology of the segments (criterion 2), yet the dorsal morphology obviously changes gradually from anterior to posterior. Furthermore, the segments of this tagma (or parts of them, see below) are very closely associated spatially (criterion 4). Tagma III forms the posterior food-processing unit. The situation of the dorsal area of this tagma is difficult to interpret. Therefore, we discuss here first the ventral details and then the dorsal side (the appendage morphology will only be touched very briefly).

**Figure 5:**
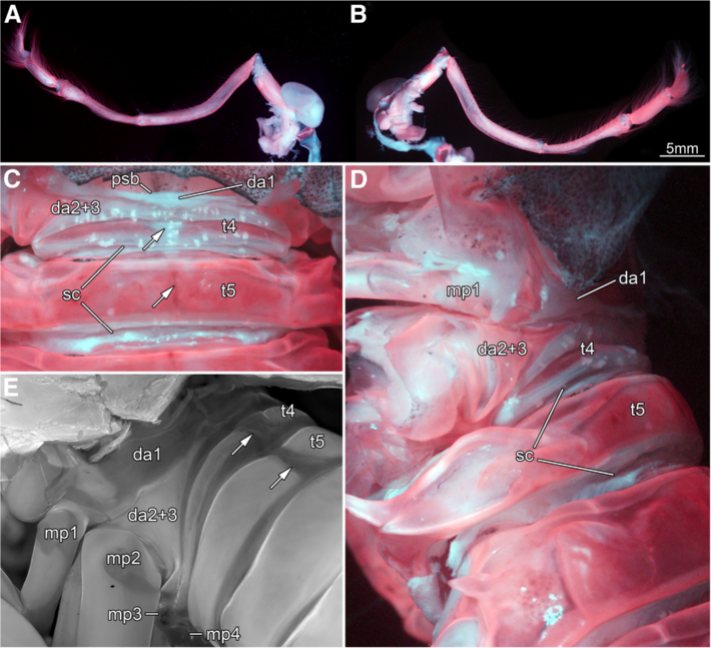
**Details of tagma III. A-D. ***Erugosquilla massavensis* (Kossmann, 1880); macro-fluorescence settings. **A**, **B**. Maxilliped 1. **A**. Posterior view. **B**. Anterior view. **C**-**E**. Dorsal and dorso-lateral view on tagma III; shield removed; not to scale. **C**. Dorsal view of trunk segments 1-5. Tergite of segment five of tagma III well developed. Lower arrow marks the distinct midline of this tergite. Tergite of trunk segment four narrow and intermittent (upper arrow). Narrow sclerites are present in the valley folds between the tergites. Dorsal area corresponding to trunk segments two and three extremely narrow and without a tergite. Dorsal area corresponding to (maxillipedal) trunk segment one extremely narrow. **D**. Dorso-lateral view of the same area as in C to ease segment correspondence. Dorsal area of first trunk segment connected to its appendage. This view allows a clear identification of the sclerites as being positioned in the valley folds. **E**. Comparable view as in D on a late larval specimen of *Pseudosquillopsis cerisii* (Roux, 1828); micro-fluorescence image. The dorsal area of trunk segment one is significantly broader than in the adult of *E. massavensis* (which are comparable in this aspect to adults of *P. cerisii*). Close connection of trunk segments two and three also well apparent. Tergite of trunk segment four still relatively broad. Tergite of trunk segment five medially intermittent comparable to tergite of trunk segment four (arrows). Abbreviations other than before: da1 = dorsal area of trunk segment 1; da2 + 3 = dorsal area of trunk segments 2 and 3; psb = posterior shield border; sc = sclerite; t = tergite.

**Figure 6:**
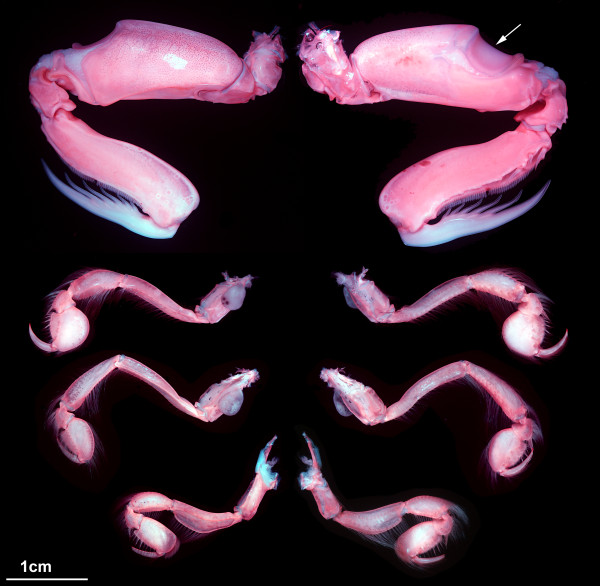
**Details of tagma III of *Erugosquilla massavensis *(Kossmann, 1880), continued.** Maxillipeds 2-5 from anterior (top; second appendage of tagma III) to posterior (bottom; fifth appendage of tagma III); macro-fluorescence settings. Arrow marks ‘meral’ saddle. Left: view from anterior, right: view from posterior.

The sternitic regions of all five segments are very short, and by this the entire region appears rather ‘squeezed’ in anterior-posterior direction (Figure 2A). All five pairs of appendages of tagma III, usually referred to as maxillipeds 1-5, are sub-chelate (Figures 5A, B, 6), and all lack an exopod, but have a lobate epipod proximo-laterally. Each appendage is composed of six elements. The exact identity of these elements is under dispute [13]. 

The first pair of appendages inserts relatively far laterally. Its sub-chelae are very small to indistinguishable (Figure 5A, B). These appendages are equipped with many fine setae and may be used, for example, to clean the eyes. Although these appendages arise from the first segment of tagma III, they curve anteriorly (laterally underneath the shield), extending well beyond tagma II to be coupled to structures of tagma I (Figure 2A).

The second appendage of tagma III, the raptorial appendage, also inserts relatively far laterally, but has a larger insertion area due to its size. It is the largest appendage of the set and bears a prominent ‘meral’ saddle on the third element (counted from proximal to distal; Figure 6). Its most distal element, the dactylus, is slightly S-shaped and equipped with five spines and a spine-like tip (Figure 6).

The following three maxillipeds (appendages 3-5 of tagma III) resemble each other in shape and size. They share the principal morphology of the raptorial appendage, but are much shorter and slimmer than it, and they lack a ‘meral’ saddle (Figure 6). Their dactyli are simply sickle-shaped and lack the spines of the dactylus of the raptorial appendage. Like the first appendage of tagma III, appendages 3-5 of tagma III are equipped with many fine setae. The insertion area of the third appendage of tagma III is situated more medially than that of the second appendage. A sclerotization around the insertion area of the third appendage of tagma III appears to be continuous with that of the second appendage, which becomes especially apparent in lateral aspect (see below; Figure 5D, E). The insertion areas of the fourth appendages of tagma III are, again, further medially located relative to those of the third appendages. The fifth appendages of tagma III insert even further medially, with very little space between their proximal parts. Posterior to their insertion areas lies an ample membranous area, which separates the legs from the main part of the sternite (Figure 2A).

For a better understanding of the dorsal area of tagma III, we prefer to describe it from posterior towards the anterior. The fifth segment of tagma III in the adult of *E. massavensis* has a well-developed tergite, which extends laterally into elongate-oval, bifid tergopleurae (Figure 5C, D). Medially on this tergite there is a shallow groove in the axial direction, also visible as a dark line (Figure 5C). In the late larval stage of *Pseudosquillopsis cerisii* this median area is not sclerotized so that the tergite consists of two parts that are not connected (Figure 5E). The tergite of segment four of tagma III in adult *E. massavensis* is here interpreted as being represented by two sclerites that are very short in anterior-posterior axis and not connected medially, so are morphologically similar to the condition in the fifth segment in the late larval stage of *P. cerisii* (Figure 5C). In the late larva of *P. cerisii*, this supposed tergite of the fourth segment of tagma III is also medially separated, closely resembling the morphology of its fifth tergite (Figure 5E).

Between the fourth and fifth tergite of tagma III and also behind the fifth tergite, sclerotic areas are developed in the valley folds between the segments in the adult of *E. massavensis* (Figure 5C, D). These sclerites should not correspond to tergites due to their position in a valley fold; they are most probably secondary sclerotizations within the connective membrane. The sclerites are not (yet?) developed in the late larval stage of *P. cerisii*. The dorsal area of the third and second segments of tagma III is very short in antero-posterior dimension (Figure 5C). The only sclerotization on these segments appears to be that of the sternitic region surrounding the insertions of the appendages, which is drawn out far dorsally, forming a large triangular area there (Figure 5C, D; da2 + 3). Segments three and two appear to share a single dorsal cuticular area, which is simply a single membranous mountain fold without sclerotization (Figure 5C). Thus, segments three and two are not only united ventrally, but also dorsally. The same arrangement was found also in the late larval specimen of *P. cerisii* (Figure 5E).

The first segment of tagma III is usually considered to be fused to the shield - so being part of tagma II (see above; [38]). Closer inspection reveals a narrow membranous area posterior to the shield, which is interpreted as representing the dorsal cuticle of this segment. The first segment of tagma III does not develop a sclerotized tergite (as segments two and three), but is represented dorsally by a membranous mountain fold anterior to that of segments two and three (Figure 5C, D). This area is very prominently developed in the late larval specimen of *P. cerisii* and is relatively broader than in the adult of *E. massavensis* (Figure 5E).

## Discussion

The detailed investigation of sclerotized and membranous areas as conducted here highlights a data set hitherto more or less neglected for taxonomic issues or in-group systematics of the stomatopod crown group (Verunipeltata). Part of these structures may already be known to experts of the taxon, but do not appear to have been specifically addressed in the literature. One main point of this paper, sclerotized versus membranous areas, cannot be simply extracted from existing publications, as there is no distinction visible in the line drawings usually provided [39,40]. Yet, this detailed knowledge is crucial for studies of stomatopods, especially of fossil ones. In these specimens, the preservation potential of (parts of) structures heavily depends on the degree of sclerotization of the animal during its lifetime (which may even vary during the molt cycle). Therefore, the present study can also help to identify and understand structures of a fossil and give hints to what kind of structures one can expect to find.

In the following, we discuss the morphology of those structures that to our knowledge differ from available data. We also review which of the morphological details we describe here have already been found among fossil stomatopods, which ones we still have to identify, and how our results will facilitate this process.

### Discussion of the morphological findings

#### The first tagma

The segments of the eyes and antennulae of stomatopods are clearly separate from the shield, which has been known for a long time [41-43]. According to our studies, these two segments are coupled with the segment of the antennae to form a tightly connected functional unit (tagma I) strictly set off from the rest of the body. This detail has not been reported before. Ventrally at least the antennular and antennal segment are connected by a large sclerite, most likely representing the anterior part of the hypostome (Figure 3A; see, e.g., [35] and discussion below). This sclerite has not been explicitly addressed in the literature and consequently its origin has not been discussed. Our interpretation as part of the hypostome is therefore the first presented hypothesis.

A hypostome is found in trilobites, agnostines [44], crustaceans [35,45], and chelicerates [46] and interpreted to be part of the euarthropod ground pattern. In various euarthropods it possesses anterior and posterior wings, encompassing the insertions of the antennulae, while the posterior wings are also the attachment sites of the antennae [35-37], similar to the present case.

Dorsally several separate sclerites are visible (Figure 3B), apparently belonging to the three segments of tagma I and resembling well-developed tergites as present in the posterior body region. The identity of the sclerites is traditionally interpreted as representing dorsal sclerites of the ocular and antennular segment [43]. This interpretation was based on the assumption that the antenna is not included in this anterior tagma. As our investigation indicates that the antennal segment also contributes to this tagma, it is more plausible that the assumed antennular sclerite is that of the antennal segment. The presumed ocular sclerite more likely represents that of the antennular segment. The ocular segment is most likely represented dorsally by the sclerotic bridge between the eye stalks.

In the ground pattern of Eucrustacea five appendage-bearing anterior body segments are incorporated into the head shield [47]. Only in rare cases within Eucrustacea, clearly secondarily evolved, the anterior body region is articulated against the head, for example, the area supposed to correspond to the antennular segment in Mystacocarida (Copepodoida, Entomostraca; [48]). More examples of such a separation of an anterior region against the rest of the body occur in certain representatives of Spelaeogriphacea and Mictacea (both Peracarida, Eumalacostraca), which bear a transverse furrow on the head shield in the area of the antennal segment, demarcating the anterior part of the shield from the rest [49,50]. In Euphausiacea (Eucarida, Eumalacostraca), both larvae and adults, the segments of eyes and antennulae can easily be removed from the rest of the head, which may also indicate that at least one of them is not part of the head shield (AM, pers. obs.; see also [51], their Figures 1, 3). Extant adult phyllocarids (Malacostraca) have a movable rostrum [52], which bears some resemblance to the movable rostral plate in stomatopods. Yet, there are no comparable examples to the situation described here, in which two anterior appendage-bearing segments have free dorsal sclerites in front of the head shield that entirely resemble the tergites posterior to the shield. The phylogenetic significance of the observation made herein cannot be estimated without data on other malacostracan or even eucrustracean species. Yet, from a functional point of view one can assume that separate tergite-like structures on each segment instead of one complete dorsal sclerotization of tagma I allow a higher movability of this anterior tagma.

#### The second tagma

The large sclerotic structure occupying most of the ventral area of tagma II is, because of the location of the mouth opening at its rear, interpreted as the posterior, extremely elongated part of the hypostome. The small lip-like structure at the posterior end of the hypostome is interpreted as the labrum, overhanging the mouth opening (Figure 4A).

In Eumalacostraca in general the distance between the antennae and the mandibles is very large, while the mouth opening is still close to the mandibles. Therefore, the morphology of the hypostome-labrum complex observed here is significantly different from that of earlier ground patterns. The entire complex is extremely elongated and appears to be bipartite: an anterior sclerotic part between the insertions of antennulae and antennae, connected by a small triangular sclerite with the strongly extended posterior part, at which end the mouth opening is overhung by the labrum. The partition of the hypostome-labrum complex into separate sclerites probably evolved because of the anterior kinesis of the head in stomatopods. With an unjointed hypostome between antennulae and the mouth opening, it would not be possible to move tagma I independently of tagma II. The evolution of this structural complex needs to be investigated further within Eumalacostraca.

#### The third tagma

The investigation of the morphology of tagma III yielded some unexpected results. One peculiar finding was that the second and third segment of this tagma (i.e., those of the large raptorial appendage and the next posterior smaller appendage) have a common sclerotization surrounding the insertions of their appendages (Figure 5C, D; da2 + 3) and also share a single dorsal cuticular area (Figure 5C). This structural unity has, to our knowledge, not been mentioned in the literature before.

Textbooks and monographs offer varying interpretations of the number of maxillipedal segments that should be incorporated into the “carapace” (shield); numbers range from one [53] to at least two [43,54] to four segments [55]. However, according to our observations none of these segments should be considered to contribute to the shield. The first segment of tagma III has its own dorsal area, recognisable through a shallow mountain fold, although its cuticle is not sclerotized (Figure 5C).

The dorsal area of the first segment of tagma III is very prominent in the larval specimen of *Pseudosquillopsis cerisii* in comparison to the adult condition in *E. massavensis* (cf. Figure 5C-E). The anterior-posterior dimension of the dorsal area of the first segment of tagma III could become reduced during ontogeny. This developmental pattern seems to occur also in the fourth segment of this tagma (compare Figure 5C and Figure 5E). As the morphological changes during the postembryonic development of *E. massavensis* have not yet been described in any publication and as there are in general only few ontogenetic sequences of stomatopods available due to the difficult breeding conditions, we think that it is justified to draw some tentative conclusions from our observations on the two different species. Yet, we are aware that we need to analyse more of the larval morphology of *E. massavensis* to confirm these interpretations.

As in segments 1-3 of tagma III in the adult of *E. massavensis,* the fourth segment of the dorsal cuticle also does not form a uniform tergite; instead, it bears a distinct but only partly sclerotized dorsal area. Only the dorsal cuticle of the fifth segment of tagma III forms a distinct tergite (Figure 5D). As this tergite is bipartite in the late larval specimen of *Pseudosquillopsis cerisii* (Figure 5E), we assume that the corresponding tergite in the adult of *E. massavensis* developed out of two sclerotic areas on either side of the dorsal midline during ontogeny (Figure 5C). The dark median line on the tergite in *E. massavensis* is interpreted by us as a developmental remnant of the closed median gap.

This finding and the subsequently more poorly developed tergites from posterior to anterior could hint of a paedomorphic effect: the tergite of the fifth segment of tagma III becomes fully developed during ontogeny, but that of the fourth segment remains bipartite in the adult, and the three further anterior segments of tagma III have no sclerotized dorsal areas at all (Figure 5C). Plesiomorphically, at least the third and fourth tergite of tagma III were fully developed in adult stomatopods, as it is visible in Carboniferous representatives [7,8]. To corroborate our assumption of a paedomorphic evolution of this feature, we have to wait for the discovery of larvae of these species with dorso-medially split tergites.

Another aspect concerning the identity of the segment of the last maxilliped is also remarkable. The appendage of this segment is ventrally closely coupled to tagma III, yet the dorsal area strongly resembles the tergites of tagma IV (not in the focus of this study, see above) and is also functionally coupled to them (see also, e.g., [42,56]). Additionally, the posterior part of the ventral area of this segment closely resembles the morphology of the succeeding ones; only the far anteriorly shifted insertion areas of the appendages are clearly incorporated into tagma III (Figure 2A). There are several examples in which segments are part of one tagma ventrally while they belong to another functional unit dorsally [47,48,57]. Yet, in our investigations the case is different, and only the anterior half of the ventral area of the segment of the last maxilliped is incorporated into one tagma, while all other parts are coupled to the other tagma. This new finding demonstrates that tagma boundaries do not necessarily have to match segment boundaries. Therefore, statements that a certain tagma consists of a certain number of segments have to be carefully re-assessed as well in other arthropods, not only to check for a dorsal-ventral tagma correspondence, but also for possible intrasegmental tagma boundaries.

### Comparisons with fossil stomatopods

#### Tagma I

**Figure 7:**
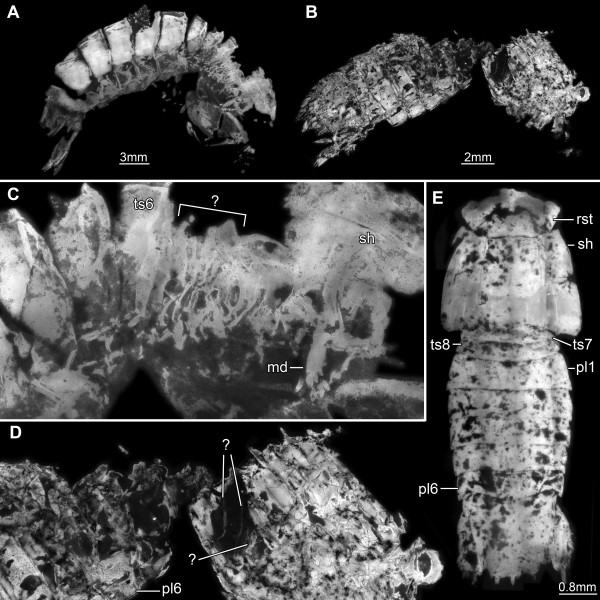
**Representatives of fossil mantis shrimps from the Jurassic (about 150 my old).** Micro-fluorescence images. Specimens exposing areas of possible interest for comparison with our new data on *Erugosquilla massavensis* (Kossmann, 1880) (for taxonomic discussion on fossil species names, see [13]). **A**, **C**. ?*Sculda pusilla*, SMNS 67505. **B**, **D**. ?*Sculda pennata/spinosa*, SMNS 63293. **A**, **B**. Overview images; anterior body end to the right. **C**, **D**. Close-up of the dorsal areas of tagma III. **C**. Mandibles are well exposed in this specimen. The condensed area of the maxillipeds makes it difficult to detect if there are still tergites developed in this region (marked with question mark). **D**. Sclerotized valley folds are possibly developed in this specimen, but the interpretation is difficult. **E**. ?*Sculda pennata/spinosa*, MB.A.528. Early juvenile specimen, demonstrating the “normal” preservation, i.e., all details of the dorsal area of functional units II and III concealed by the shield. Abbreviations other than before: pl = pleomere; sh = shield; ts = trunk segment.

Fossil stomatopods have preserved several morphological features of the first tagma in quite some detail, while other features remain obscure. For example, eyes are frequently preserved (Figure 7B, D) [12,14], but never in structural detail, i.e., exhibiting the differentiation of the visual surface (lobes, midband) or ommatidia, although co-occurring malacostracan fossils have preserved ommatidia (e.g., isopods; [58]). Even the well-sclerotized proximal eye region, the eye stalk, could not be identified in any fossil form. Much better preserved are the antennulae and antennae [13,14]. Of the antennulae, their peduncle and the exact pattern of the flagellar arrangement as well as the total number of the flagellar annuli have been described from Mesozoic mantis shrimps [12-14]. Of the antennae, the flagellate endopod and the bipartite exopod including its fine setation are known [12,14]. Until now no details could be identified of the sclerotic ventral and dorsal elements of tagma I. While the movable rostrum is easily identifiable (Figure 7E) [13], neither the tergites of the antennular and antennal segments have yet been found, nor the sclerotic bridge between the eyes, the anterior hypostome part, or the antero-ventral sclerite. 

#### Tagma II

The large hypostome, although well-sclerotized in the extant species, and the associated labrum have not yet been identified in any fossil stomatopod. In laterally and dorsally preserved specimens this region is usually covered by the well-sclerotized shield (Figure 7E). The hypostome may be recognized only from a ventral orientation. Yet, even in specimens preserved well enough that the hypostome should be present, it is concealed by the maxillipeds, which are folded onto the body ventrally - a position that is also typical for modern mantis shrimps.

The mouthparts are also almost unknown from the fossil representatives. The mandible has been found in a single Mesozoic specimen, which had the shield broken off in that area (Figure 7A, C) [13]. In some of the Carboniferous representatives the mandibles can be recognized as an impression through the shield, but are never free [8]. Similar impressions might be present among the Mesozoic forms; a mandibular palp could not be identified in any of the fossil forms. Paragnaths and the two pairs of maxillae have also never been found in any fossil mantis shrimp. The sclerotization pattern of these structures, as we documented it here, is important for future search of these structures in the fossil representatives. It may well be that of the maxillulae only the sclerotized lobes are preserved, maybe even just their slightly better sclerotized outer rims, whereas the soft intermediate cuticular regions are unlikely to be preserved.

Sternites of tagma II may have been preserved in some of the laterally preserved specimens, however, a clear identification is very difficult. The sternites are usually folded medially and then superimposed on each other. While the larger sternites of the pleon still retain some identifiable structures when preserved like this, the small and highly condensed sternites of the mandibular, maxillular and maxillary segments simply end up as an unsorted accumulation of small sclerites between remains of the appendages and maybe even covered by parts of the shield.

#### Tagma III

Remarkably, the first sub-chelate appendage of tagma III, the grooming appendage in modern forms, has not been found in any of the fossil stomatopod specimens. The succeeding four pairs of maxillipeds are well-documented for many different species from Mesozoic and Palaeozoic strata [6,13]. The median stacking of the maxillipeds can be deduced from their insertion areas in the specimens of the Mesozoic species and can be assumed for most of the Carboniferous material, although many of these specimens are preserved laterally.

However, the sternitic and tergitic situation in tagma III is not fully understood in the fossils and appears to differ in certain aspects from that of the modern forms. Some of the Carboniferous stomatopods possess well-developed tergites on the third and fourth segment of tagma III [7,8]. For the Mesozoic species this is less clear. The segments are already highly condensed and it is unclear which of these segments still has developed tergites (Figure 7C). Some of these fossils might also have had the narrow sclerotic elements in the valley folds as described above for *Erugosquilla massavensis* (Figure 7B, D). It is furthermore unclear whether appendages two and three of this tagma were already forming a single unit in some of these fossil forms.

## Conclusions

The pattern of tagmosis of extant stomatopods, as re-described here, differs in several aspects from that commonly described in the literature. Major points of divergence between our data and previous observations or interpretations are:

The segments of eyes, antennulae and antennae form a tightly packed functional unit, tagma I (i.e., not only those of eyes and antennulae).

The first post-maxillary segment (that of the grooming appendage) is not incorporated into the head shield (feature of tagma II), but retains its own unsclerotized dorsal area within tagma III (i.e., there is no cephalothorax present).

The segments of the maxillipeds two and three (second and third segment of tagma III) lack tergites, but share a single, very short unsclerotized dorsal area separate from the head shield. The ventral sclerotization surrounding the insertion area of the second maxilliped appears to be continuous with that surrounding the insertion area of the third maxilliped.

The tergite of the segment of the fourth maxilliped (fourth segment of tagma III) is very short and appears bipartite due to an unsclerotized median area. The interpretation of this bipartite condition as the result of paedomorphic heterochrony is based on two observations: 1) The corresponding tergite is fully developed in adult stomatopods from the Carboniferous. 2) In late larval stages of extant stomatopods also the tergite of the segment of the fifth maxilliped (fifth segment of tagma III) is bipartite, but becomes a continuous tergite in later stages.

Most of these details have not yet been identified in fossil stomatopods. Therefore, this study may serve as a guideline on which structures might be detectable in fossil stomatopods. In this way it may be possible to identify the specific character states that are less derived from the ground pattern of Stomatopoda than those in extant species, which again facilitates the reconstruction of a more detailed evolutionary scenario. Studies similar to this one on other representatives of Malacostraca would be very helpful in amending our current approach.

## Material and methods

The studied material of *Erugosquilla massavensis* was collected from Port Said harbour, the northern entrance of the Suez Canal on the Egyptian Mediterranean coast. Fresh specimens were obtained from the catch of trawling vessels and immediately preserved in 70% ethanol. The material of *Pseudosquillopsis cerisii* was provided by Björn van Reumont, Bonn, and collected by Nils Brenke, Wilhelmshaven, at the Great Meteor Seamount, Northeast Atlantic, during the Meteor Expedition M42/3. Comparative fossil material was loaned from the collections of the Staatliches Museum für Naturkunde Stuttgart (SMNS) and the Museum für Naturkunde Berlin (MB.A).

### Dissection

Extant specimens were immersed in 70% ethanol and carefully dissected with the aid of fine pincers, needles and knives. All parts of the specimens were stored in 70% ethanol and also documented directly within this fluid (cf. Figure 1).

### Macrophotography using crossed polarized filters

The camera used for documentation was a Canon EOS 450D with either a MP-E 65 mm lens or an EF-S 60 mm lens, depending on the size of the documented structures. Some specimens or parts of these were documented under crossed polarized light [59]. Using crossed polarized light reduces reflections on the liquid surface and enhances the contrast significantly [32,33]. Additionally, the light settings were very even and almost undirected, i.e., they are less likely to produce artefacts, such as shadows or glittering.

### Macro-fluorescence photography and fluorescence microscopy

Macro-fluorescence settings and fluorescence microscopy settings follow those described in [33]. For macro-photography the same camera settings were used as described above. Under these fluorescence conditions membranous areas appear white, while sclerotized areas appear pink. An exception are heavily sclerotized areas (e.g., mandibular edges, dactyli of maxillipeds), which are also white under fluorescence. For fluorescence microscopy documentation of *Pseudosquillopsis cerisii* we used a Zeiss Axioskop 2 equipped with an AxioCam; for details see [12,31,60].

### Image processing

Image enhancing was performed with image fusion (CombineZM, Image Analyzer) and image stitching (Adobe Photoshop CS3, Microsoft Image Composite Editor), or - as a combination of these methods - composite imaging [12,31,60]. Histogram adjustments of the enhanced images were done in Gimp or Photoshop CS3. Dirt particles in the background were manually removed in some cases.

## Competing interests

The authors declare that they have no competing interests.

## Authors’ contributions

WSS collected the studied material of *Erugosquilla massavensis*. JTH dissected and documented the extant material. CH documented the fossil material. CH and JTH processed the images. All authors participated in the morphological and evolutionary interpretations, drafted the manuscript, read and approved it.
